# Serum Concentrations of Benzaldehyde, Isopentanaldehyde and Sex Hormones: Evidence from the National Health and Nutrition Examination Survey

**DOI:** 10.3390/toxics11070573

**Published:** 2023-06-30

**Authors:** Zhilei Mao, Rui Yuan, Xu Wang, Kaipeng Xie, Bo Xu

**Affiliations:** 1 Changzhou Maternal and Child Healthcare Hospital, Changzhou Medical Center, Nanjing Medical University, Changzhou 213000, China; 2 Department of Endocrinology, Children’s Hospital of Nanjing Medical University, Nanjing 210008, China; 3 Department of Public Health, Nanjing Maternity and Child Health Care Hospital, Women’s Hospital of Nanjing Medical University, Nanjing 210004, China; 4 State Key Laboratory of Reproductive Medicine, School of Public Health, Nanjing Medical University, Nanjing 211166, China

**Keywords:** benzaldehyde, isopentanaldehyde, sex hormones, NHANES

## Abstract

Exposure to environmental chemicals could disturb the balance of sex hormones. However, the studies on Benzaldehyde, Isopentanaldehyde exposure and sex hormones are still limited. Based on the data of 1064 participants in the National Health and Nutrition Examination Survey (NHANES), we used the linear regression model and restricted cubic spline (RCS) model to evaluate the associations of Benzaldehyde/Isopentanaldehyde exposure with testosterone (TT), estradiol (E2), sex hormone binding globulin (SHBG), free androgen index (FAI) and the ratio of TT to E2 (TT/E2). A ln-unit increase in Benzaldehyde was associated with lower TT (β = −0.048, *P* = 0.030) and E2 (β = −0.094, *P* = 0.046) in all participants. After further adjustment for menopausal status, Benzaldehyde was negatively associated with E2 (β = −0.174, *P* = 0.045) in females. The interaction between Benzaldehyde and gender was significant (*P*_inter_ = 0.031). However, Isopentanaldehyde showed a positive association with SHBG and TT/E2 in all participants (all *P* < 0.05). The positive associations of Isopentanaldehyde with TT, SHBG and TT/E2 were found in males but not in females. RCS plots illustrated the linear associations of Benzaldehyde with E2 (*P*_non-linear_ = 0.05) in females and Isopentanaldehyde with TT (*P*_non-linear_ = 0.07) and TT/E2 (*P*_non-linear_ = 0.350) in males. The non-linear relationships were identified between Isopentanaldehyde and SHBG in males (*P*_non-linear_ = 0.035). Our findings indicated the effects of Benzaldehyde and Isopentanaldehyde exposure on sex hormones, and the effects had the gender specificity. Cohort studies and high-quality in vitro and in vivo experiments are needed to confirm the specific effects and uncover the underlying mechanisms.

## 1. Introduction

Sex steroid hormones are a series of important molecules including testosterone (TT), estradiol (E2) and sex hormone binding globulin (SHBG). TT, an androgenic steroid, plays a key role in spermatogenesis and male fertility [1]. E2, the primary female sex hormone, is responsible for ovarian quality and reproductive aging in females. SHBG, a transport protein for testosterone and estradiol in the blood [2]. Generally, sex hormones have a broad range of regulative function in both females and males. Of note, a growing body of studies showed that dysregulation of sex hormones were involved in multiple diseases, such as abnormal folliculogenesis, polycystic ovary syndrome [3], hormone-dependent tumors [4], diabetes [5], cardiovascular [6], immunity and autoimmune disease [7], metabolic syndrome [8], neurological and cognitive functioning [9], etc. Recent studies suggested that serum TT concentrations are associated with cause-specific mortality and health span termination [10,11]. Sex hormones disturbances and their associated diseases burden become major issues of public health concern. Therefore, studies regarding the contributing factors of sex hormones disturbances could provide an opportunity for preventing these chronic diseases.

It is known that genetic predisposition [12], smoking status [13] and nutrition [14] have influences on sex hormones. Emerging evidence showed that exposure to environmental chemicals might disturb the balance of sex hormones. Aldehydes are a class of organic compounds characterized by the appearance of a −HC = O. Apart from exogenous sources including tobacco and e-cigarettes smoke, wood burning, occupational exposure, food additives and cosmetic products [15,16], aldehydes can be generated endogenously by cellular metabolic pathways, such as lipid peroxidation, carbohydrate or metabolic ascorbic acid autoxidation, amine oxidases, etc. [15]. Due to aldehydes having potentially carcinogenic and mutagenic effects [17], adverse health concerns have been raised. Several epidemiology reports have revealed the relationships between aldehydes and health outcomes, such as diabetes mellitus [18], obesity [19], low bone mineral density and high osteopenia/osteoporosis [20], etc. However, studies on aldehydes and sex hormones are limited.

Serum concentrations of aldehydes were measured in the population of the National Health and Nutrition Examination Survey (NHANES) from 2013 to 2014. Silva et al. reported that the detection rate of Benzaldehyde, Isopentanaldehyde and Hexanaldehyde were approximately above 95% in 1843 participants aged 12 years or older in the NHANES [21]. Benzaldehyde is a kind of unsaturated aldehyde, whereas Isopentanaldehyde and hexanaldehyde are saturated aldehydes. Recently, Xing et al. assessed the associations between six aldehydes with high detective rate (>80%) and sex hormones in 851 participants [22]. It is worth noting that the number of missing values of baseline characteristics including BMI, education and smoking status increases with the number of analyzed aldehydes. Meanwhile, the sample size decreased sharply when analyzing multiple aldehydes together. Additionally, they did not assess the effect of aldehydes on free androgen index (FAI) and the ratio of TT to E2 (TT/E2). FAI serves as a good clinical indicator of hypertension status in menopause [23] and a marker for the severity of hyperan-drogenemia [24]. TT/E2 reflects the activity of aromatase and the relative balance of TT and E2 in the circulation [25]. It also plays an important role in spermatogenesis and fertilization ability [26].

Therefore, considering the increasing missing values of Hexanaldehyde and baseline characteristics result from analyzing Benzaldehyde, Hexanaldehyde and Isopentanaldehyde together, and the potentially clinical utility of FAI and TT/E2 in the population, we evaluate the associations of serum Benzaldehyde/Isopentanaldehyde and sex hormones including TT, E2, SHBG, FAI and TT/E2 in NHANES adults with complete variable information.

## 2. Materials and Methods

### 2.1. Study Design and Population

NHANES is a continuing cross-sectional survey aiming to assess the nutrition and health status of the U.S. population. A new round of investigations is carried out every two years. Questionnaires, physical examination and laboratory examination were collected. Surveys have been approved by the Research Ethics Review Board of the Centers for Disease Control and Prevention (CDC). All the participants have provided informed consents.

**Figure 1:**
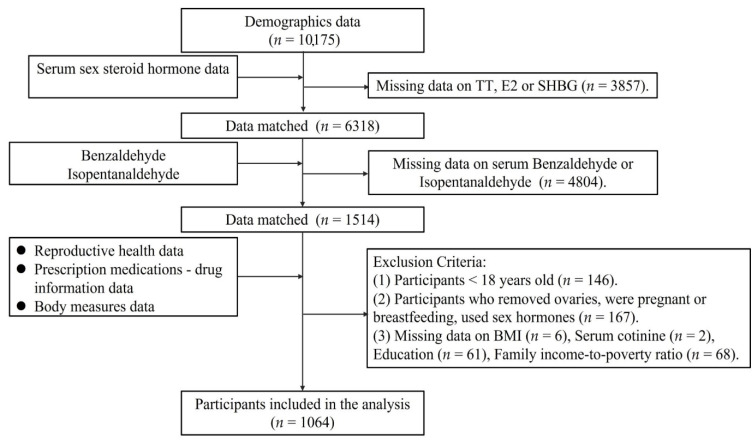
Flow chart of the study participant selection.

In the current study, we utilized data from NHANES 2013 to 2014 cycle. A total of 10,175 participants were surveyed, the participants (*n* = 1514) with accessible data on Benzaldehyde, Isopentanaldehyde and sex hormones were matched. Participants under the age of 18 were excluded, because of their immaturity of the hypothalamic-pituitary-gonadal (HPG) axis. Women who had their ovaries removed, were pregnant or breastfeeding and participants with sex hormones usage were excluded. Participants with missing covariate values (BMI, Serum cotinine, Education, Family income-to-poverty ratio) were also excluded. Finally, 1064 participants were included for our analysis. The flowchart is presented in Figure 1.

### 2.2. Measurement of Serum Benzaldehyde and Isopentanaldehyde

The measurements, detection methods and quality control of aldehydes in serum are available on the NHANES website Laboratory Procedure Manual [27]. We selected two aldehydes (Benzaldehyde and Isopentanaldehyde) in our further analyses. Limits of detection (LOD) were 0.461 ng/mL for Benzaldehyde and 0.119 ng/mL for Isopentanaldehyde. The concentrations lower than LOD were replaced by LOD/√2.

### 2.3. Measurement of Serum Sex Hormones

Three sex hormones (TT, E2, SHBG) in serums were measured. TT and E2 were detected by isotope dilution liquid chromatography tandem mass spectrometry (ID-LC-MS/MS) method [28], the SHBG was tested by Sex Hormone-Binding Globulin Immunoassay [29]. The LODs were 0.75 ng/mL, 2.994 pg/mL and 0.800 nmol/L for TT, E2 and SHBG, respectively. We also constructed FAI, calculated as (TT/SHBG) × 100 and the TT/E2.

### 2.4. Covariates

Potential covariates including age, gender, ethnicity, Body mass index (BMI), smoking status, educational levels and the family income to poverty ratio were collected. Family income was categorized into 3 levels: low income (≤1.3), medium income (>1.3 to 3.5) and high income (>3.5) [30]. BMI was calculated from the data of physical examination, which was divided into three levels: normal weight (<25 kg/m^2^), overweight (25–30 kg/m^2^) and obese (≥30 kg/m^2^). Serum cotinine levels were grouped into two sets: cotinine < LOD and cotinine ≥ LOD. We also adjusted the seasons and the timing of the blood draw as these might induce possible bias.

### 2.5. Statistical Analysis

As serum concentrations of Benzaldehyde, Isopentanaldehyde, and sex hormones did not fit a normal distribution, continuous variables were expressed as medians (interquartile range, IQR) and categorical variables were expressed as numbers (percentages). The baseline characteristics between males and females were compared by wilcoxon signed-rank tests for continuous variables or chi-squared tests for categorical variables. Specifically, we divided Benzaldehyde and Isopentanaldehyde exposure levels into tertiles, and compared the differences by χ^2^ tests and post hoc tests for categorical variables, Kruskal–Wallis tests and Dunn’s tests for multiple comparisons were employed for continuous variables.

The concentrations of Benzaldehyde, Isopentanaldehyde and sex hormones were ln-transformed to reduce the skewness in the distributions. Multiple linear regression models were applied to estimate the relationships between Benzaldehyde, Isopentanaldehyde and sex hormones. The coefficients are presented as β values and 95% confidence intervals (CIs). In categorical analyses, β values and 95% CIs were evaluated by comparing the second and third tertile groups with the first tertile. We also regarded exposure tertiles as continuous variables to calculate the *P* values for trend. A restricted cubic spline (RCS) model was used to curve the dose–response relationships between Benzaldehyde, Isopentanaldehyde and sex hormones. Moreover, we also explored the effect stratified by selected characteristics (gender, age and smoking status) in the associations of Benzaldehyde, Isopentanaldehyde exposure with sex hormones. All regression models were performed after adjusting for age, gender, BMI, race, education, serum cotinine, family income-to-poverty ratio, six-month time period, time of blood draw, and menopausal status (in females). R software version 4.2.1 was used for statistical analyses. *P* < 0.05 of both sided tests was considered as statistical significance.

## 3. Results

### 3.1. Characteristics of Participants

The baseline characteristics of study population were presented in Supplementary Table S1. A total of 1064 participants were enrolled in our study, including 599 males and 465 females. Overall, the study population had a median age of 46 years, 47.74% of them were non-Hispanic White, 68.89% were overweight/obese (BMI ≥ 25). More than half (75.38%) of the participants had a high detection rate of serum cotinine, a high educational level of college or above (53.57%), a low family income-to-poverty ration (39.85%) and 51.41% were examined on May 1 through October 31. The overall detectable rate was 93.42% for Benzaldehyde and 99.34% for Isopentanaldehyde in our analyzed population. For exposure, the median (IQR) concentrations of Benzaldehyde and Isopentanaldehyde were 1.30 (0.80–1.90) ng/mL and 0.54 (0.35–1.11) ng/mL, respectively. For sex hormones, the median (IQR) concentrations of TT, E2, SHBG, FAI and TT/E2 ratio were 230.50 (23.60–425.00) ng/dL, 24.50 (15.40–37.60) pg/mL, 47.74 (31.99–72.68) nmol/L, 20.37 (1.36–36.20) and 105.46 (9.37–185.97), respectively.

### 3.2. Distributions of Characteristics according to the Tertile Groups of Benzaldehyde and Isopentanaldehyde

As shown in Table 1, the distributions of ethnicity, serum cotinine, education and family income-to-poverty ratio between three Benzaldehyde tertile groups were significant (all *P* < 0.05). The median concentrations of TT and E2 in participants with the highest Benzaldehyde tertile group were significantly lower than participants with the lowest Benzaldehyde tertile group (*P* = 0.031, *P* = 0.010, respectively). No significant differences were found in serum SHBG, FAI and TT/E2 levels among different Benzaldehyde tertile groups.

The distributions of age, gender, ethnicity, BMI, serum cotinine, education, family income-to-poverty ratio and time of blood draw between three Isopentanaldehyde tertile groups were significant (all *P* < 0.05, Table 2). For sex hormones, the median concentrations of TT, SHBG, TT/E2 in participants with highest Isopentanaldehyde tertile group were significantly higher than participants with the lowest tertile group (all *P* < 0.001, Table 2). The median concentrations of FAI in the second tertile group were significantly higher than that in the first tertile group (Table 2). No significant difference was found in serum E2 among different Isopentanaldehyde tertile groups.

### 3.3. Associations of Benzaldehyde Exposure with Sex Hormones Levels

**Figure 2:**
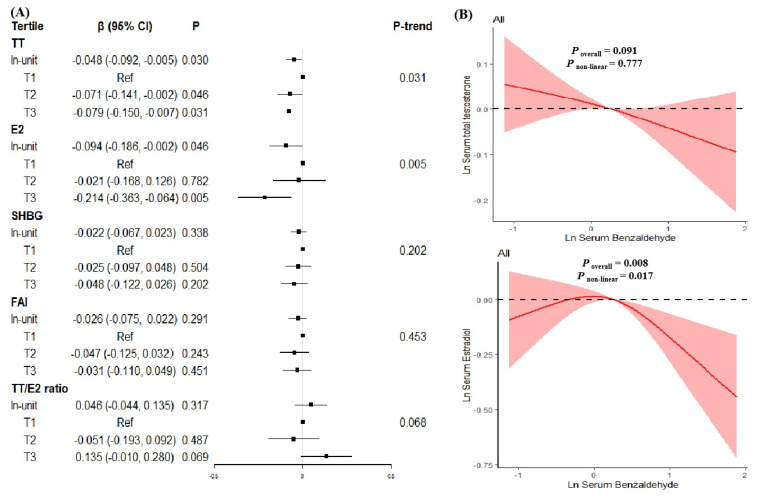
The relationships between serum Benzaldehyde levels and serum sex hormones levels in all participants. (**A**) β (95%CI) in serum sex hormones levels associated with serum Benzaldehyde in continuous and categorical analyses. The black square in the center of the line is a β value. The width of the line represents the 95% CI. (**B**) Dose–response relationship between serum Benzaldehyde levels and serum total testosterone levels (upper) and serum estradiol levels (lower) in all participants.

The associations of Benzaldehyde concentrations with sex hormones levels in all participants are shown in Figure 2. After adjustment for age, gender, BMI, ethnicity, education, serum cotinine, family income-to-poverty ratio, six-month time period and time of blood draw, a ln-unit increase in Benzaldehyde was associated with lower TT (β = −0.048, *P* = 0.030) and E2 (β = −0.094, *P* = 0.046) in all participants. When modeling serum Benzaldehyde and levels as a categorical variable, TT and E2 levels decreased as the tertiles of Benzaldehyde increased in all participants (*P*_trend_ = 0.031, 0.005, respectively). After adjusting for confounders, the exposure-response relationship showed that Benzaldehyde exhibited a linear association with TT (*P* for nonlinearity = 0.777), but a non-linear association with E2 (*P* for nonlinearity = 0.017).

**Figure 3:**
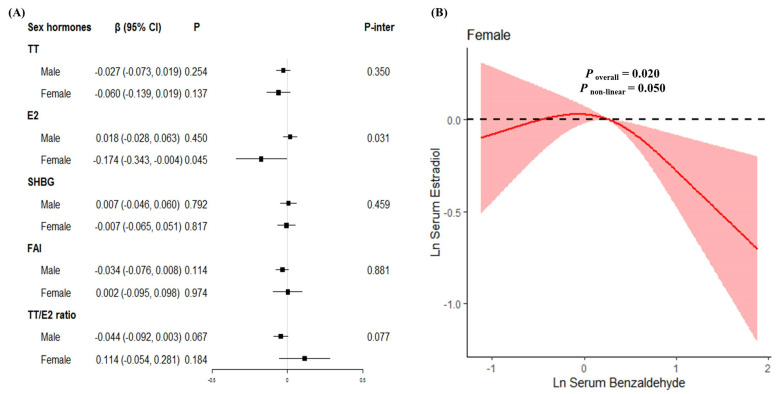
The relationships between serum Benzaldehyde levels and serum sex hormones levels in continuous analysis stratified by gender. (**A**) β (95%CI) in serum sex hormones levels associated with serum Benzaldehyde in male participants and female participants. The black square in the center of the line is a β value. The width of the line represents the 95% CI. (**B**) Dose–response relationship between serum Benzaldehyde levels and serum estradiol levels in females.

We further explored the associations of Benzaldehyde exposure with sex hormones stratified by gender, age and serum cotinine in subgroups analyses. In continuous analyses, no significant association was observed in males after adjustment for age, BMI, race, education, serum cotinine, family income-to-poverty ratio, six-month time period and time of blood draw (Figure 3A). After further adjustment for menopausal status, we observed that Benzaldehyde was marginally associated with E2 (β = −0.174, *P* = 0.045) in females. The interaction between Benzaldehyde and gender was significant (*P*_inter_ = 0.031). A linear association was observed in the RCS model (*P* for nonlinearity = 0.050, Figure 3B). Subgroup analyses by age and serum cotinine groups showed no significant associations between Benzaldehyde exposure and sex hormones (Supplementary Tables S2 and S3).

### 3.4. Associations of Isopentanaldehyde Exposure with Sex Hormones

**Figure 4:**
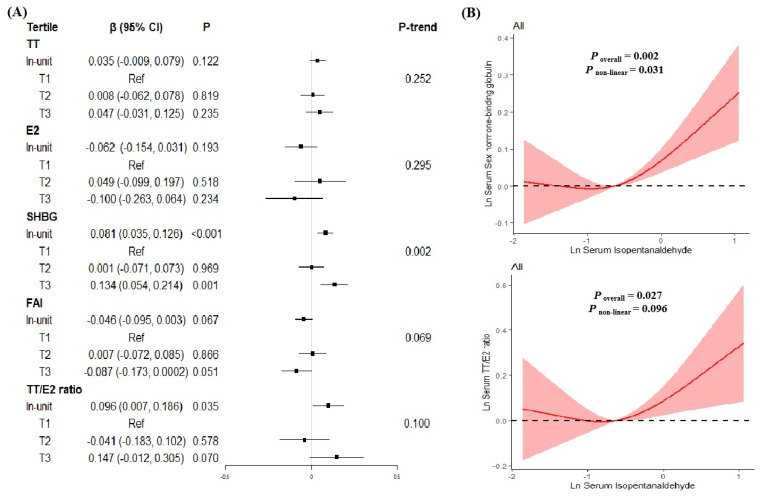
The relationships between serum Isopentanaldehyde levels and serum sex hormones levels in all participants. (**A**) β (95%CI) in serum sex hormones levels associated with serum Isopentanaldehyde in continuous and categorical analyses. The black square in the center of the line is a β value. The width of the line represents the 95% CI. (**B**) Dose–response relationship between serum Isopentanaldehyde levels and serum SHBG levels (upper) and serum TT/E2 ratio (lower) in all participants.

As shown in Figure 4, Isopentanaldehyde exposure showed positive associations with SHBG and TT/E2 in all participants (β_SHBG_ = 0.081, *P* < 0.001; β_TT/E2_ = 0.096, *P* = 0.035) after adjustment for confounders. The same trend between Isopentanaldehyde exposure and SHBG was observed when serum Isopentanaldehyde levels serve as a categorical variable (*P*_trend_ = 0.002). Compared to the lowest Isopentanaldehyde tertile group, we observed a positive association in highest tertile group (β = 0.134, *P* = 0.001). Further RCS results indicated that a non-linear association with SHBG (*P* for nonlinearity = 0.031), but a linear association of Isopentanaldehyde exposure with TT/E2 ratio (*P* for nonlinearity = 0.096).

**Figure 5:**
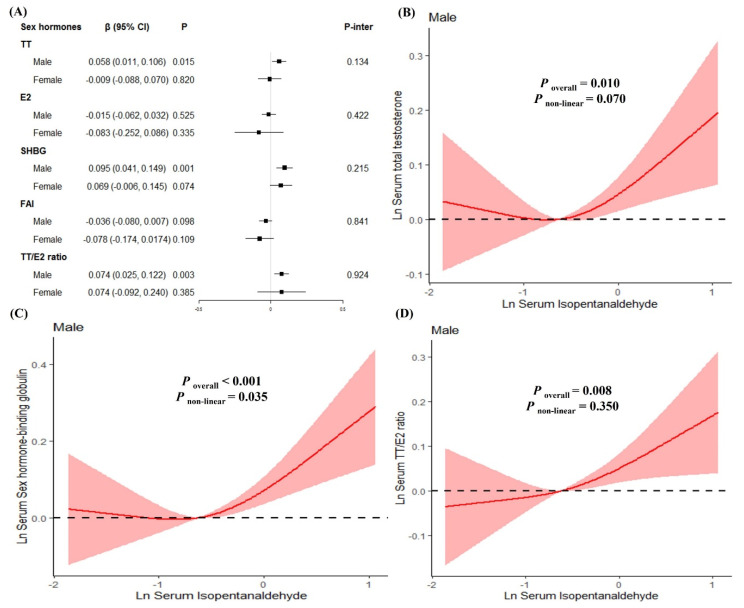
The relationships between serum Isopentanaldehyde levels and serum sex hormones levels stratified by gender. (**A**) β (95%CI) in serum sex hormones levels associated with serum Isopentanaldehyde in male participants and female participants. (**B**) Dose–response relationships between serum Isopentanaldehyde levels and serum total testosterone levels in males. (**C**) Dose–response relationships between serum Isopentanaldehyde levels and serum sex hormone binding globulin levels in males. (**D**) Dose–response relationships between serum Isopentanaldehyde levels and serum TT/E2 ratio in males.

The positive associations of Isopentanaldehyde with TT, SHBG and TT/E2 were observed in males (β_TT_ = 0.058, *P*_TT_ = 0.015; β_SHBG_ = 0.095, *P*_SHBG_ = 0.001; β_TT/E2_ = 0.074, *P*_TT/E2_ = 0.003; Figure 5A). However, no significant associations were found in females. In RCS models, Isopentanaldehyde had a linear negative association with TT and TT/E2 ratio in males (all *P* for nonlinearity > 0.05, Figure 5B,D), and non-linear associations with SHBG (*P* for nonlinearity = 0.035) in males (Figure 5C).

Further stratification by gender groups (Supplementary Table S2) demonstrated that Isopentanaldehyde was positively associated with SHBG (β = 0.090, *P* = 0.005) and negatively associated with FAI (β = −0.074, *P* = 0.040) in younger participants. The positive association of Isopentanaldehyde with TT was found in older participants (β = 0.076, *P* = 0.031). Stratifications by serum cotinine groups showed that Isopentanaldehyde was positively associated with SHBG and TT/E2 ratioin participants with serum cotinine ≥ LOD (β_SHBG_ = 0.090, *P*_SHBG_ < 0.001; β_TT/E2_ = 0.111, *P*_TT/E2_ = 0.021; Supplementary Table S3).

## 4. Discussion

Our study found that Benzaldehyde exposure was negatively associated with TT and E2 in all participants. Benzaldehyde was also negatively associated with E2 in females. We observed the significant interactive effects between Benzaldehyde exposure and gender. On the contrary, Isopentanaldehyde showed positive associations with SHBG and TT/E2 in all participants. The positive associations of Isopentanaldehyde with TT, SHBG and TT/E2 were found in males, but not in females. When modeling serum Benzaldehyde and Isopentanaldehyde levels as categorical variables, similar results were verified. Moreover, we found the linear associations of Benzaldehyde with E2 in females and Isopentanaldehyde with TT and TT/E2 in males, as illustrated in RCS plots. We identified the non-linear relationships between Isopentanaldehyde and SHBG in males. 

Our study found different effects of Benzaldehyde and Isopentanaldehyde on sex hormones. As for structure, aldehydes can be categorized into saturated aldehydes or unsaturated aldehydes by whether unsaturated bonds exist other than the aldehyde functional group. Benzaldehyde is a kind of unsaturated aldehyde, while Isopentanaldehyde is a kind of saturated aldehyde. It is probably because of the different structures between Benzaldehyde and Isopentanaldehyde that their effects on sex hormones are varies [18]. Based on the fact that aldehydes are volatile, have a high reactivity and complex detection, studies on the relationships between exposure of Benzaldehyde, Isopentanaldehyde and sex hormones are scarce. Several studies showed that aldehydes have cytotoxic, mutagenic, genotoxic, carcinogenic effects [31] and reproductive toxicity [32]. Sex hormones are required for the development and function of reproductive organs [33]. However, due to the limited population study, available evidence on the associations between aldehydes exposure and sex hormones were based on the few in vitro studies, which showed that serum TT levels were decreased in rats after formaldehyde exposure [34].

The possible mechanisms underlying the associations between exposure to Benzaldehyde, Isopentanaldehyde and sex hormones were unclear. Previous studies showed that formaldehyde exposure might exert ROS-mediated oxidative damage [35], and ROS could affect male sex hormone levels and disrupt the hormonal balance, which could regulate male reproductive functions [36]. Other studies displayed that increased heat shock protein (HSP) synthesis was detected after formaldehyde exposure in rat pups [37]. HSPs are a group of highly conserved proteins with diverse functions. The loss of Hsp27 could specifically prevent TT-induced rapid signaling, and the bidirectional interplay of Hsp27 and estrogen receptors ensured the function of E2 [38]. In addition, previous research showed that formaldehyde could influence DNA methylation in sperm [39]. Other evidence suggested that DNA methylation was associated with estrogen in healthy postmenopausal women [40]. In a word, ROS-mediated oxidative damage, the change of HSPs and DNA methylation might be the mechanisms underlying the associations between exposure to Benzaldehyde, Isopentanaldehyde and sex hormones. However, high-quality in vitro and in vivo experiments are needed to reveal the different effects of Benzaldehyde and Isopentanaldehyde on sex hormones.

Our study had several strengths. Our study evaluated the relationships between aldehydes and sex hormones using NHANES, which included a large sample size, broad age group and national representation. In addition to adjusting for common potential confounders, we considered potential bias factors such as seasons, the timing of the blood draw and the menstrual phase in our linear regression models. As there was a potential association between smoking and sex hormones [13,41,42], we assessed the smoking status of each sample by serum cotinine. However, there were some limitations. Firstly, because our research was a cross-sectional survey, it was unable to demonstrate the causality relationship. Secondly, we only evaluated the association of Benzaldehyde/Isopentanaldehyde exposure with sex hormones and did not further assess exposure, sex hormones and a specific disease via mediation analysis to uncover the mediator role of sex hormones. Finally, the participants of our study were from the United States, other nationally representative studies are necessary to validate our results.

Our findings gain novel insight into the potential health effects of Benzaldehyde/Isopentanaldehyde on sex hormones. Further studies are warranted to explore the potential mechanisms underlying the associations and the implementation of strategies to prevent adverse health effects.

## Figures and Tables

**Table 1:** Characteristics of study participants according to the tertiles of Benzaldehyde levels.

Characteristics	Benzaldehyde, ng/mL			
	Tertile1 (*n* = 354)	Tertile2 (*n* = 360)	Tertile3 (*n* = 350)	*P*
	<0.961	0.961–1.680	>1.680	
Age, years, median (IQR)	45 (35–60)	45 (32–59)	47 (34.25–59.75)	0.605
Gender, *n* (%)				0.394
Male	209 (59.04%)	201 (55.83%)	189 (54.00%)	
Female	145 (40.96%)	159 (44.17%)	161 (46.00%)	
Ethnicity, *n* (%)				0.005
Mexican Americans	38 (10.73%) ^a^	40 (11.11%) ^a^	63 (18.00%) ^b^	
Other Hispanic	28 (7.91%) ^a^	30 (8.33%) ^a^	19 (5.43%) ^a^	
Non-Hispanic White	176 (49.72%) ^a^	169 (46.94%) ^a^	163 (46.57%) ^a^	
Non-Hispanic Black	76 (21.47%) ^a^	70 (19.44%) ^a,b^	49 (14.00%) ^b^	
Others	36 (10.17%) ^a^	51 (14.17%) ^a,b^	56 (16.00%) ^b^	
BMI, kg/m^2^, median (IQR)	27.80 (24.60–32.58)	27.50 (23.60–32.50)	27.60 (23.75–32.15)	0.575
<25, *n* (%)	99 (27.97%)	117 (32.50%)	115 (32.86%)	0.554
25.0–30.0, *n* (%)	125 (35.31%)	111 (30.83%)	111 (31.71%)	
≥30.0, *n* (%)	130 (36.72%)	132 (36.67%)	124 (35.43%)	
Serum Cotinine				<0.001
≥LOD	292 (82.49%) ^a^	272 (75.56%) ^a,b^	238 (68.00%) ^b^	
<LOD	62 (17.51%)	88 (24.44%)	112 (32.00%)	
Education, *n* (%)				0.003
Less than high school	105 (29.66%) ^a^	72 (20.00%) ^b^	73 (20.86%) ^b^	
High school or equivalent	88 (24.86%) ^a^	82 (22.78%) ^a^	74 (21.14%) ^a^	
College or above	161 (45.48%) ^a^	206 (57.22%) ^b^	203 (58.00%) ^b^	
Family income-to-poverty ratio, *n* (%)				0.003
Low	169 (47.74%) ^a^	132 (36.67%) ^b^	123 (35.14%) ^b^	
Medium	110 (31.07%) ^a^	121 (33.61%) ^a^	119 (34.00%) ^a^	
High	75 (21.19%) ^a^	107 (29.72%) ^b^	108 (30.86%) ^b^	
Six-month time period				0.068
November 1 through April 30	187 (52.82%)	159 (44.17%)	171 (48.86%)	
May 1 through October 31	167 (47.18%)	201 (55.83%)	179 (51.14%)	
Time of blood draw				0.129
Morning	166 (46.89%)	180 (50.00%)	156 (44.57%)	
Afternoon	125 (35.31%)	133 (36.94%)	148 (42.29%)	
Evening	63 (17.8%)	47 (13.06%)	46 (13.14%)	
Sex hormones, median (IQR)				
TT,ng/dL	274.90 (26.18–446.75) ^a^	223.58 (23.21–407.89) ^a,b^	207.50 (20.11–396.50) ^b^	0.031
E2, pg/mL	25.60 (16.50–39.50) ^a^	25.15 (16.43- 40.83) ^a^	22.50 (13.93–32.78) ^b^	0.010
SHBG, nmol/L	49.68 (32.24–71.99)	47.25 (32.08–73.36)	45.49 (31.43–72.31)	0.727
FAI	23.91 (1.49–36.22)	19.15 (1.44–34.88)	18.07 (1.19–36.48)	0.298
TT/E2 ratio	114.60 (8.85–196.19)	99.31 (7.12–176.30)	98.41 (13.72–180.50)	0.264

^a,b^ Groups with the same superscript letters are not significantly different. *P* values for comparison over all three categories. Abbreviations: IQR, interquartile range; BMI, body mass index; LOD, limit of detection; TT, total testosterone; E2, estradiol; SHBG, sex hormone-binding globulin; FAI, Free androgen index.

**Table 2:** Characteristics of study participants according to the tertiles of Isopentanaldehyde levels.

Characteristics	Isopentanaldehyde, ng/mL			
	Tertile1 (*n* = 354)	Tertile2 (*n* = 355)	Tertile3 (*n* = 355)	*P*
	<0.391	0.391–0.861	>0.861	
Age, years, median (IQR)	45 (34–61) ^a,b^	47 (35–63) ^a^	45 (33–56) ^b^	0.048
Gender, *n* (%)				
Male	168 (47.46%) ^a^	219 (61.69%) ^b^	212 (59.72%) ^b^	<0.001
Female	186 (52.54%)	136 (38.31%)	143 (40.28%)	
Ethnicity, *n* (%)				<0.001
Mexican Americans	63 (17.8%) ^a^	59 (16.62%) ^a^	19 (5.35%) ^b^	
Other Hispanic	32 (9.04%) ^a^	27 (7.61%) ^a,b^	18 (5.07%) ^b^	
Non-Hispanic White	140 (39.55%) ^a^	168 (47.32%) ^b^	200 (56.34%) ^c^	
Non-Hispanic Black	60 (16.95%) ^a^	51 (14.37%) ^a^	84 (23.66%) ^b^	
Others	59 (16.67%) ^a^	50 (14.08%) ^a,b^	34 (9.58%) ^b^	
BMI, kg/m^2^, median (IQR)	28.20 (24.73–34.18) ^a^	28.10 (24.90–32.80) ^a^	26.70 (22.70–30.55) ^b^	<0.001
<25, *n* (%)	99 (27.97%) ^a^	91 (25.63%) ^a^	141 (39.72%) ^b^	<0.001
25.0–30.0, *n* (%)	101 (28.53%) ^a^	125 (35.21%) ^a^	121 (34.08%) ^a^	
≥30.0, *n* (%)	154 (43.5%) ^a^	139 (39.15%) ^a^	93 (26.2%) ^b^	
Serum Cotinine				<0.001
≥LOD	226 (63.84%) ^a^	230 (64.79%) ^a^	346 (97.46%) ^b^	
<LOD	128 (36.16%)	125 (35.21%)	9 (2.54%)	
Education, *n* (%)				<0.001
Less than high school	77 (21.75%) ^a^	67 (18.87%) ^a^	106 (29.86%) ^b^	
High school or equivalent	69 (19.49%) ^a^	72 (20.28%) ^a^	103 (29.01%) ^b^	
College or above	208 (58.76%) ^a^	216 (60.85%) ^a^	146 (41.13%) ^b^	
Family income-to-poverty ratio, *n* (%)				<0.001
Low	118 (33.33%) ^a^	110 (30.99%) ^a^	196 (55.21%) ^b^	
Medium	123 (34.75%) ^a^	116 (32.68%) ^a^	111 (31.27%) ^a^	
High	113 (31.92%) ^a^	129 (36.34%) ^a^	48 (13.52%) ^b^	
Six-month time period				0.443
November 1 through April 30	172 (48.59%)	181 (50.99%)	164 (46.2%)	
May 1 through October 31	182 (51.41%)	174 (49.01%)	191 (53.8%)	
Time of blood draw				0.020
Morning	189 (53.39%) ^a^	168 (47.32%) ^a,b^	145 (40.85%) ^b^	
Afternoon	116 (32.77%) ^a^	138 (38.87%) ^a,b^	152 (42.82%) ^b^	
Evening	49 (13.84%) ^a^	49 (13.8%) ^a^	58 (16.34%) ^a^	
Sex hormones, median (IQR)				
TT,ng/dL	55.99 (19.75–380.75) ^a^	256.79 (26.30–405.50) ^b^	284.00 (25.18–499.84) ^b^	<0.001
E2, pg/mL	25.40 (15.35–45.93)	24.20 (16.85–34.45)	23.00 (14.60–34.85)	0.142
SHBG, nmol/L	45.97 (29.01–69.61) ^a^	44.61 (29.96–66.22) ^a^	51.98 (36.30–79.33) ^b^	<0.001
FAI	5.72 (1.18–35.33) ^a^	23.72 (1.59–36.41) ^b^	21.92 (1.43–36.24) ^a,b^	0.022
TT/E2 ratio	61.55 (5.67–157.61) ^a^	116.79 (12.21–178.99) ^b^	130.67 (21.02–216.30) ^b^	<0.001

^a,b,c^ Groups with the same superscript letters are not significantly different. *P* values for comparison over all three categories. Abbreviations: IQR, interquartile range; BMI, body mass index; LOD, limit of detection; TT, total testosterone; E2, estradiol; SHBG, sex hormone-binding globulin; FAI, Free androgen index.

## Data Availability

The NHANES data is publicly available online.

## Supplementary Materials

The following supporting information can be downloaded at: https://www.mdpi.com/article/10.3390/toxics11070573/s1, Table S1: Baseline characteristics of study participants, Table S2: Associations of serum Benzaldehyde and Isopentanaldehyde with sex hormones in all participants according to different age groups, Table S3: Associations of serum Benzaldehyde and Isopentanaldehyde with sex hormones in all participants according to different serum cotinine groups.
