# Supplementary material for: Serum Concentrations of Benzaldehyde, Isopentanaldehyde and Sex Hormones: Evidence from the National Health and Nutrition Examination Survey
Source: Toxics. 2023 Jun 30;11(7):573. doi: 10.3390/toxics11070573 (PMC10383974; doi:10.3390/toxics11070573)
Supplement: Supplementary file 1 [file toxics-11-00573-s001.zip › toxics-2365046-supplementary.pdf]

toxics

MDPI

Supplementary Materials: Serum Concentrations of Benzaldehyde, Isopentanaldehyde and Sex Hormones: Evidence from the National Health and Nutrition Examination Survey

Zhilei Mao, Rui Yuan, Xu Wang, Kaipeng Xie and Bo Xu

Table S1. Baseline characteristics of study participants.

Characteristics	All<br>*n* = 1064
Age, year, median (IQR)	46 (34-60)
Ethnicity, n (%)	
Mexican Americans	141 (13.25%)
Other Hispanic	77 (7.24%)
Non-Hispanic White	508 (47.74%)
Non-Hispanic Black	195 (18.33%)
Others	143 (13.44%)
BMI, kg/m^2, n (%)	27.6 (23.9-32.5)
<25	331 (31.11%)
25.0–30.0	347 (32.61%)
>30.0	386 (36.28%)
Serum Cotinine	
≥LOD	802 (75.38%)
<LOD	262 (24.62%)
Education, n (%)	
Less than high school	250 (23.5%)
High school or equivalent	244 (22.93%)
College or above	570 (53.57%)
Family income-to-poverty ratio, n (%)	
Low	424 (39.85%)
Medium	350 (32.89%)
High	290 (27.26%)
Six-month time period	
November 1 through April 30	517 (48.59%)
May 1 through October 31	547 (51.41%)
Time of blood draw	
Morning	502 (47.18%)
Afternoon	406 (38.16%)
Evening	156 (14.66%)
Menopausal status*	
Premenopausal	234
Postmenopausal	174
Missing	57
Aldehydes, ng/mL, median (IQR)	
Benzaldehyde	1.30 (0.80-1.90)
Isopentanaldehyde	0.54 (0.35-1.11)
Sex hormones, median (IQR)	
TT,ng/dL	230.50 (23.60-425.00)
E2, pg/ml	24.50 (15.40-37.60)
SHBG, nmol/L	47.74 (31.99-72.68)
FAI	20.37 (1.36-36.20)

TT/E2 ratio	105.46 (9.37-185.97)

Abbreviations: IQR, interquartile range; BMI, body mass index; LOD, limit of detection; TT, total testosterone; E2, estradiol; SHBG, sex hormone-binding globlin; FAI, Free androgen index.
* The menopausal status in females.

Table S2. Associations of serum Benzaldehyde and Isopentanaldehyde with sex hormones in all participants according to different age groups.

Exposure	Sex hormones	<50 (**n** = 600)			≥50 (**n** = 464)		
		β [a]	(95% CI)[a]	*P*[a]	β[a]	(95% CI)[a]	*P*[a]
Benzaldehyde	TT	-0.044	(-0.103, 0.014)	0.140	-0.040	(-0.106, 0.026)	0.233
	E2	-0.085	(-0.190, 0.020)	0.113	-0.009	(-0.114, 0.095)	0.862
	SHBG	-0.026	(-0.091, 0.038)	0.427	0.000	(-0.063, 0.063)	0.995
	FAI	-0.018	(-0.091, 0.054)	0.622	-0.040	(-0.109, 0.029)	0.258
	TT/E2 ratio	0.040	(-0.063, 0.144)	0.445	-0.031	(-0.132, 0.070)	0.549
Isopentanaldehyde	TT	0.016	(-0.042, 0.073)	0.598	0.076	(0.007, 0.146)	0.031
	E2	-0.040	(-0.143, 0.063)	0.442	-0.017	(-0.128, 0.093)	0.761
	SHBG	0.090	(0.027, 0.153)	0.005	0.063	(-0.004, 0.130)	0.064
	FAI	-0.074	(-0.145, -0.004)	0.040	0.013	(-0.060, 0.087)	0.721
	TT/E2 ratio	0.056	(-0.045, 0.157)	0.280	0.094	(-0.013, 0.200)	0.085

[a] Adjusted for gender, BMI, race, education, serum cotinine, family income-to-poverty ratio, six-month time period and time of blood draw.

Table S3. Associations of serum Benzaldehyde and Isopentanaldehyde with sex hormones in all participants according to different serum cotinine groups.

Exposure	Sex hormones	≥LOD			<LOD		
		β[a]	(95% CI)[a]	*P*[a]	β[a]	(95% CI)[a]	*P*[a]
Benzaldehyde							
	TT	-0.051	(-0.103, 0.0005)	0.053	-0.048	(-0.130, 0.035)	0.256
	E2	-0.105	(-0.209, -0.001)	0.048	-0.117	(-0.316, 0.082)	0.252
	SHBG	-0.036	(-0.087, 0.016)	0.173	0.019	(-0.074, 0.112)	0.683
	FAI	-0.015	(-0.071, 0.040)	0.584	-0.067	(-0.169, 0.035)	0.197
	TT/E2 ratio	0.054	(-0.048, 0.156)	0.301	0.069	(-0.115, 0.253)	0.464
Isopentanaldehyde							
	TT	0.038	(-0.010, 0.089)	0.119	0.056	(-0.090, 0.202)	0.451
	E2	-0.073	(-0.169, 0.023)	0.136	0.064	(-0.29, 0.417)	0.725
	SHBG	0.090	(0.043, 0.137)	<0.001	-0.035	(-0.200, 0.130)	0.677
	FAI	-0.052	(-0.103, -0.001)	0.047	0.091	(-0.089, 0.272)	0.323
	TT/E2 ratio	0.111	(0.017, 0.206)	0.021	-0.007	(-0.334, 0.319)	0.965

[a] Adjusted for gender, BMI, race, education, serum cotinine, family income-to-poverty ratio, six-month time period and time of blood draw.
